# Adulteration and Contamination of Commercial Sap of* Hymenaea* Species

**DOI:** 10.1155/2017/1919474

**Published:** 2017-02-20

**Authors:** Katyuce de Souza Farias, Sarah Alves Auharek, Andréa Luiza Cunha-Laura, Jeana Mara Escher de Souza, Geraldo Alves Damasceno-Junior, Mônica Cristina Toffoli-Kadri, Wander Fernando de Oliveira Filiú, Edson dos Anjos dos Santos, Marilene Rodrigues Chang, Carlos Alexandre Carollo

**Affiliations:** ^1^Laboratório de Produtos Naturais e Espectrometria de Massas, UFMS, Cidade Universitária, Campo Grande, MS, Brazil; ^2^Laboratório de Pesquisa em Bioensaios, UFMS, Cidade Universitária, Campo Grande, MS, Brazil; ^3^Laboratório de Botânica, UFMS, Cidade Universitária, Campo Grande, MS, Brazil; ^4^Laboratório de Biofisiofarmacologia, UFMS, Cidade Universitária, Campo Grande, MS, Brazil; ^5^Laboratório de Bioquímica Clínica, UFMS, Cidade Universitária, Campo Grande, MS, Brazil; ^6^Laboratório de Bioquímica, UFMS, Cidade Universitária, Campo Grande, MS, Brazil; ^7^Laboratório de Pesquisas Microbiológicas, UFMS, Cidade Universitária, Campo Grande, MS, Brazil

## Abstract

The* Hymenaea stigonocarpa* and* Hymenaea martiana* species, commonly known as “jatobá,” produce a sap which is extracted by perforation of the trunk and is commonly used in folk medicine as a tonic. For this study, the authenticity of commercial samples of jatobá was verified by the identification of the main compounds and multivariate analysis and contamination by microbial presence analysis. The acute toxicity of the authentic jatobá sap was also evaluated. The metabolites composition and multivariate analysis revealed that none of the commercial samples were authentic. In the microbiological contamination analysis, five of the six commercial samples showed positive cultures within the range of 1,700–100,000 CFU/mL and the authentic sap produced no signs of toxicity, and from a histological point of view, there was the maintenance of tissue integrity. In brief, the commercial samples were deemed inappropriate for consumption and represent a danger to the population.

## 1. Introduction

The herbal drugs used by the population are sold on the street and local markets where they are, in general, not appropriately identified or packaged [1], and this restrained relationship between trade and quality can directly affect the health of the population [2].

Zaroni et al. [3] have shown in a survey carried out in the south of Brazil that 79% of the medicinal plants produced in the region exhibit high counts of aerobic microorganisms, molds, and yeasts. Another study, about the quality of commercial samples of guaco* (Mikania glomerata)* in the central market and pharmacies of Belo Horizonte, Brazil, showed a huge difference in the concentration of coumarin (a chemical marker of this species), which can result in variations of the normal pharmacological activity [4]. In India, a study showed that 84% of the “churna” samples (a formulation widely used by the local population as an herbal medicine) contain mercury, lead, and cadmium higher than those considered as acceptable by the World Health Organization [5].

Developed countries are stricter in monitoring this sector. In an investigation on the quality of commercial dried spices and herbs sold in the UK, it was found that 96% of the 2,833 samples sold were within the quality standards required and that only 1.5% presented some form of the pathogenic agent, like* Salmonella* spp. [6].

Another factor that may influence the safety of medicinal plants is toxicity. Many traditional herbs are potentially dangerous [7, 8]. It is common to find reports on ingestion or exposure to toxic plants throughout the literature, whether accidental or due to errors during the processing of the plant extracts. Vichova and Jahodar [9] reported a study performed in pediatric hospitals in the Czech Republic between 1996 and 2001. During this period there were 174 cases of accidental exposure to toxic species by patients from 0 to 18 years, where the group of 1 to 3-year-olds was the most affected, with 42.3% of the cases. What stands out within the research is the fact that the identified toxic species are readily available, found in public parks, school yards, gardens, and inside homes.

The genus* Hymenaea* (Leguminosae) is distributed throughout India, Africa, Central America, and many parts of South America [10]. This genus is widely used in popular medicine, particularly for treating inflammations, rheumatisms, coughs, and anemia [11, 12].

The species* Hymenaea* have a particular use in folk medicine when the sap of the trunk is used as a tonic [13, 14]. Also, the stem bark of* H. stigonocarpa* has antidiarrhetic, gastroprotective and healing effect on gastric and duodenal ulcers [15, 16], as well as antitermitic and antioxidant effect [17]. Among the compounds identified in this species, the terpenes of the epi-labdanoid type, present in the resin, were highlighted [18]. The bark of* H. martiana* showed antioxidant activity and the presence of phenol compounds [19], in addition to antinociceptive, antiedematogenic, anti-inflammatory, and analgesic properties [20], and antimicrobial agents acting against* Cryptococcus neoformans*,* Trichophyton rubrum, Trichophyton mentagrophytes,* and* Microsporum canis* [21].* H. courbaril* (also known as jatobá), a substitute for* H. stigonocarpa* and* H. martiana,* is also widely used to treat wounds, bronchitis, and stomach diseases [22].

Despite the widespread use of the species in folk medicine and the broad distribution, there are no studies in the literature which evaluate the quality controls of commercial sap or methods able to check their authenticity. Thus, the present study has evaluated commercial samples of jatobá sap concerning the presence of microbiological contamination, followed by authenticity tests, as well as the evaluation of acute toxicity of an authentic sap.

## 2. Materials and Methods

### 2.1. Acquisition and Processing of the Plant Material

Six samples of jatobá sap (Js-1 to Js-6) sold in Campo Grande, Mato Grosso do Sul, Brazil, were obtained. Authentic sap have been obtained from the* H. martiana* Hayne (named Hm-1) and the* H. stigonocarpa* Mart. Ex Hayne species (denominated Hs-1 and Hs-2). Authentic samples were collected in Serra do Amolar (Mato Grosso do Sul, Brazil) by perforating the trunk. Stem bark was also collected (named Hm-sb1 from* H. martiana* and Hs-sb1 and Hs-sb2 from* H. stigonocarpa*). Species were identified by Geraldo Alves Damasceno-Junior and vouchers of the plants were deposited at the CGMS Herbarium under the numbers 38077 and 38078, respectively.  Table 1 shown the samples, places, and code identifications used during this work. Hm-sb1, Hs-sb1, and Hs-sb2 were extracted with boiling water for 30 minutes (decoction). They were then lyophilized, as were all of the evaluated samples. The samples were kept refrigerated until the beginning of the analyses. Since the chemical profiles of the control sap were similar and the* H. stigonocarpa* was the most commonly used, Hs-1 was selected for a toxicity analysis as well as used as a control sample during the microbiological contamination test.

### 2.2. Authenticity

The commercial and authentic samples were evaluated at the University of São Paulo, School of Pharmaceutical Sciences of Ribeirão Preto, using a high-performance liquid chromatograph coupled with a micrOTOF II high-resolution mass spectrometer (Bruker) (HPLC-MS) equipped with a C-18 column (Sigma-Aldrich, ODS-2, 5 um, 4.6 × 250 mm). The method includes the use of water containing 1% acetic acid (phase A) and acetonitrile containing 1% acetic acid (phase B) with the following gradient: for three minutes, 3% of B, followed by a gradient of 3–12% of B for 25 min, 12–16% of B for 20 min, and 16–100% of B for three minutes. The column was washed and restabilized for seven minutes before the next injection. The flow used was 1 mL/min. All samples were injected in a random order and the same concentration of 1 mg/mL. MS spectra were acquired in positive mode and for the metabolomic analysis; the mass signals obtained from raw data files were extracted and aligned within the metAlign software program, with a final alignment of 5592 mass signals. Subsequently, the signals belonging to the same molecules, isotopes, fragments, and adducts, were regrouped using the MSClust software, resulting in 67 reconstructed metabolites. The molecular formulas of the major compounds were determined by peaks obtained by the mass spectrometer, taking into account the elements C, H, O, and N, the error being equal to or less than 5 ppm. The UV spectrum also characterized the compounds. Catechin and quercetin were identified through comparison with the authenticity standard (Sigma). Finally, the data was analyzed in Metaboanalyst 3.0 platform using Principal Component Analysis (PCA) and Heatmap. For PCA, data was log average transformed. For Heatmap, dispersion profile of the compounds and hierarchical clustering was organized using Euclidean distance, Ward clustering algorithm, and the top 40 metabolites selected by ANOVA (*p* ≤ 0.05).

### 2.3. Microbiological Evaluation

The methodology was in accordance with the United States Pharmacopeia [23] with minor modifications. Enrichment broths and selective media were used to research the viable microorganism count as well as for the identification of* Salmonella sp.*,* Escherichia coli*,* Staphylococcus aureus*,* Pseudomonas aeruginosa*, and* Candida albicans* [24]. Every procedure was carried out aseptically and in triplicate in a biosafety cabinet. For the counting of the colony forming unit (CFU) an automated colony counter was used (model Q295B) (Quimis, Brazil), and the data was expressed as a mean average ± standard deviation. The analysis following the recommendations set out by the World Health Organization [25].

### 2.4. Acute Toxicity

45 Wistar rats of the species* Rattus norvegicus* were acquired from the animal facility of the Federal University of Mato Grosso do Sul (23 males and 22 females) with an average weight of 283 ± 13 grams. The animals underwent an adaptation period of 10 days, housed throughout the experiment in plastic cages equipped with beds of selected sawdust, with a light/dark cycle of 12 hours, with water and food* at libitum.* The animals were divided into four groups. The doses were administered using intragastric gavages in amounts of 1000, 2500, and 5000 mg/kg of the sample Hs-1. The control group received only water. The acute toxicity protocol was followed according to the recommendations of the Organization for Economic Cooperation and Development [26] and those of the specific resolution of the National Agency for Sanitary Surveillance [27]. All procedures and protocols followed approved guidelines for the ethical treatment of animals, according to the Ethics Committee in Animal Experimentation from the Federal University of Mato Grosso do Sul (Protocol # 460/2012).

After the administration the different doses of jatobá sap, the animals were evaluated in the first half-hour and then two hours after the administration of the intragastric gavages samples and then every twelve hours for 14 days. The activity and coordination of the motor system and reflexes and the activity of the central and autonomous nervous systems such as general activity, sensory analysis (vocal tremor, irritability, reflex headset, touch response, response to tail pinch and corneal reflex), psychomotor analysis (contortion, position of the hind, righting reflex, body tone, and grip strength), analysis of the central nervous system (tremors, convulsions, tail erection, sedation, anaesthesia, and ataxia), and analysis of the autonomic nervous system (ptosis, presence of lacrimation, urination, defecation, cyanosis, and hypothermia) were evaluated. Differences were observed in body weight and food and water consumption before and after the experiment.

After the 14-day period, the animals were anesthetized with a ketamine-xylazine solution and then euthanized. The bowel was displaced and the aorta exposed to collect blood in two tubes (one containing an anticoagulant to determine the hematological parameters and one without for the extraction of serum and to determine the biochemical parameters). Subsequently, the kidneys and liver were removed, weighed, evaluated macroscopically, and processed for histopathological analysis. In brief, representative and extracted fragments were fixed using Bouin's solution. Once fixed, the tissue fragments were dehydrated, cleared, and embedded in paraffin wax. The samples were cut into 5 *μ*m thick sections and stained with haematoxylin-eosin for histological analyses.

### 2.5. Biochemical and Hematological Parameters

For the analysis of hematological parameters, the values of erythrocytes, leukocytes, platelets, hemoglobin, hematocrit, mean corpuscular volume (MCV), mean corpuscular hemoglobin (MCH), and mean corpuscular hemoglobin concentration (MCHC) were measured using an automated hematology analyzer model Sp-100i (SYSMEX, Kobe, Japan). The differential leukocyte count was performed on each device and confirmed in blood smears stained with May-Grunwald-Giemsa (100 cells analyzed and counted in each smear). For the biochemical analysis, blood samples were centrifuged at 1026 ×g and the levels of glucose, urea, creatinine, alanine aminotransferase (ALT) and aspartate aminotransferase (AST), amylase, and lipase were determined. The tests were performed at the Clinical Laboratory of the University Hospital of the Federal University of Mato Grosso do Sul using an automated analyzer (brand COBAS 6000, Switzerland). All analyses were performed by monitoring of the control serum provided by the Excellence Program of Medical Laboratories (PELM) of Controlab, Brazilian Society of Clinical Pathology, Rio de Janeiro, and the control serum from the College of American Pathologists, Northfield, IL, USA.

### 2.6. Histomorphometry of the Liver and Kidney

The individual volume of the hepatocytes was obtained from their nuclear volume and the proportion between nucleus and cytoplasm. To calculate these proportions, a 540-point square lattice was placed over the sectioned materials at 1000x magnification. At least four thousand points of hepatocytes were counted for each animal (*n* = 8 in each group). Because the hepatocyte nucleus in rats is spherical, its nucleus volume was obtained to our knowledge of the mean nuclear diameter. For this purpose, the diameters of forty nuclei were measured for each animal. Hepatocyte nuclear volume was expressed in *μ*m^3^ and obtained using the formula 4/3*πr*3, where *r* = nuclear diameter/2 [28]. In the kidney, the same analyses were performed, and the nuclear volume of the cells of the proximal convoluted tubule was estimated. Moreover, the diameters of the renal and glomerular corpuscles were also measured.

### 2.7. Statistical Analysis

The results of the acute toxicity evaluation, as well as the biochemical, hematological, and histological parameters, were expressed as a mean average ± standard deviation. A variance analysis (ANOVA) was used, of a route followed by Tukey's multiple range test using the GraphPad Prism 4®* software*. The values for *p* less than 0.05 were considered significant.

## 3. Results and Discussion

An important part of the quality control is the confirmation of authenticity. Some species, even though being similar taxonomically, may have different pharmacological and toxicological effects [29].

**Figure 1:**
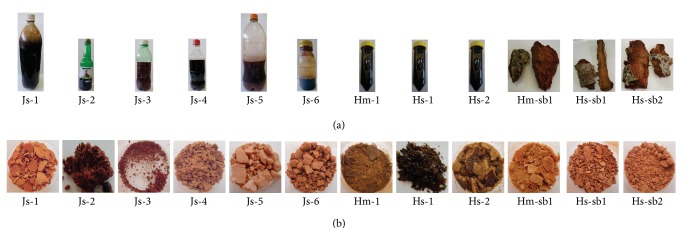
(a) Samples in the packaging in which they were acquired and (b) dry residues obtained after lyophilization. The authentic sap is listed as Hm-1, Hs-1, and Hs-2 and those obtained by decoction of the stem bark as Hm-sb1, Hs-sb1, and Hs-sb2. Commercial samples are listed as Js-1-Js-6. Samples Hs-1, Hs-2, Hs-sb1, and Hs-sb2 belong to* H. stigonocarpa* and Hm-1 and Hm-sb1 to* H. martiana*.

Regarding organoleptic traits, the samples exhibited visual and yields differences (Figure 1): 1.47% (Js-1), 1.48% (Js-2), 0.52% (Js-3), 1.21% (Js-4), 2.24% (Js-5), 1.11% (Js-6), 2.92% (Hm-1), 6.84% (Hs-1), 4.06% (Hs-2), 4.75% (Hm-sb1), 2.17% (Hs-sb1), and 3.54% (Hs-sb2).

Even the dried mass of control samples (Hm-1, Hs-1, and Hs-2) was inconstant, which may reflect different concentrations of compounds. This variation may be related to the age of the plant species or other edaphoclimatic factors. Specific studies are required to determine the best parameters for the collecting of the sap. The commercial samples (Js-1, Js-2, Js-3, Js-4, Js-5, and Js-6) exhibited significantly lower values than the control (*t*-test *p* less than 0.05), which may suggest dilution.

The quality control of the plant samples using traditional techniques is not always a suitable alternative because plant species have various phytochemicals that act together. Thus, chromatographic* fingerprinting* may be able to describe the complexity of the plant extract [30–32] and help characterize those plant extracts that do not have any preestablished monographs.

**Figure 2:**
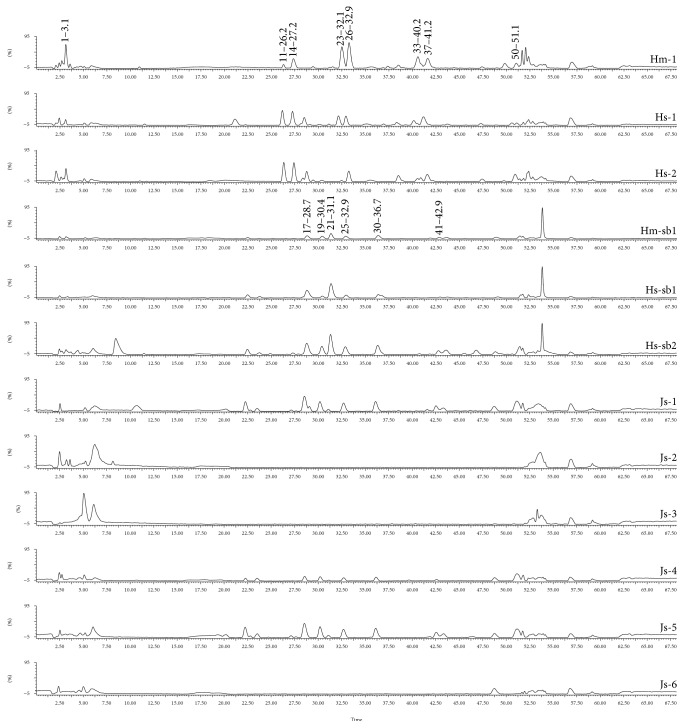
Chromatograms obtained using HPLC-MS of the control sap, Hm-1, Hs-1, and Hs-2 of the bark decoctions Hm-sb1, Hs-sb1, and Hs-sb2, and of the commercial samples Js-1 to Js-6. The samples Hm-1 and Hm-sb1 belong to* H. martiana* and Hs-1, Hs-2, Hs-sb1, and Hs-sb2 to* H. stigonocarpa.* The main peaks of the sap and stem bark are also shown in the chromatogram. Table 2 shows detailed information about the peaks.

The chromatograms of the samples acquired by HPLC-MS are shown in Figure 2.

Among the compounds found in the control sap and jatobá stem bark, the main components were flavonoids and procyanidins, characterized by the molecular formula and the UV spectrum and compared to referential data. Despite the lack of studies performed on jatobá sap, these classes of compounds have been described in the wood bark of this plant species [17]. About the compounds found in the bark used for the present study; only procyanidins have been previously described in the literature on* H. courbaril,* the substitute species for* H. stigonocarpa* [33].

**Figure 3:**
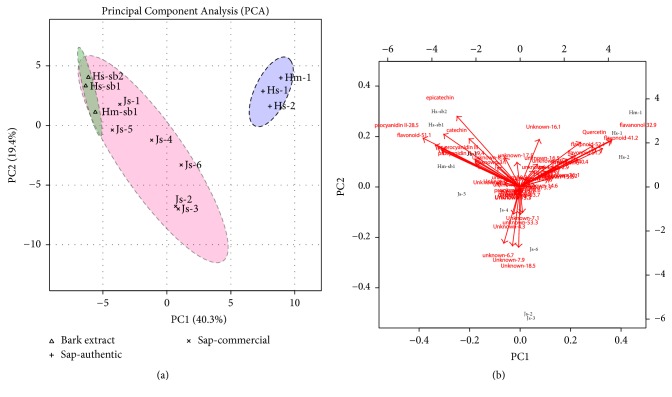
(a) Principal Component Analysis (PCA) and (b) Biplot of loadings and samples. Js-1 to Js-6 (commercial samples), Hm-1, Hs-1, and Hs-2 (authentic jatobá sap), and Hm-sb1, Hs-sb1, and Hs-sb2 (bark decoctions). The samples Hm-1 and Hm-sb1 belong to* H. martiana* and Hs-1, Hs-2, Hs-sb1, and Hs-sb2 to* H. stigonocarpa.* Ellipses represent 95% confidence area around the groups.

**Figure 4:**
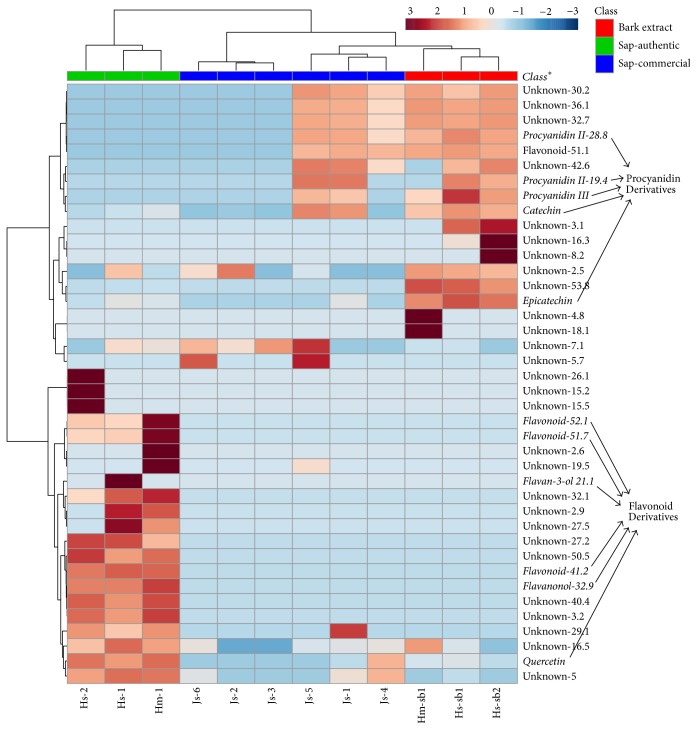
Heatmap of peaks from jatobá sap samples. The legend on the superior part indicates the class of the sample. The legend on the bottom indicates the sample identification (Table 1). The values have been log transformed and hierarchical clustering was organized using Euclidean distance, Ward clustering algorithm, and the top 40 metabolites selected by ANOVA (*p* ≤ 0.05). Red color indicates higher concentration; blue color indicates lower concentration. ^*∗*^More details about the compounds shown in the right border can be found in Table 2.

There is a clear distinction between commercial samples and controls sap. Considering that there are no Pharmacopeia monographs of this plant species, the authenticity analysis took into consideration the main peaks of authentic samples and discriminant analysis by PCA, Biplot (Figure 3), and Heatmap (Figure 4). The main peaks of the samples from* H. stigonocarpa* Hs-1 and Hs-sb1 are shown in Table 2.

PCA, Biplot, and Heatmap analysis reveal the presence of two main groups. After evaluation and comparison, the main peaks characteristic of Hm-1, Hs-1, and Hs-2 are the compounds 1, 11, 14, 23, 26, 33, 37, and 50 (Table 2) and found no resemblance to the commercial samples. Among the commercial and stem bark samples, two groups were formed one by Js-2, Js-3, and Js-6 pooled separately and other by Js-1, Js-4, Js-5, Hm-sb1, Hs-sb1, and Hs-sb2 (Figure 4). The main peaks for these groups were 17, 19, 24, 25, 30, and 41. The separation of these groups could be caused by a different dilution of commercial samples, as observed in mass spectrometry chromatograms (Figure 2).

The PCA (Figure 3(a)) represents 59.7% of the variability of the data, where the components 1 and 2 represent 40.3% and 19.4%, respectively. The Heatmap and Biplot showed clearly a separation based on metabolites of the analyzed samples. Commercial and stem bark sample are similar in the composition mainly by the presence of catechin, epicatechin, and procyanidin derivatives, while the presence of flavonoids as quercetin distinguishes the sap from both species (Figures 3(b) and 4). The present study demonstrates a simple and efficient method for the screening of the counterfeiting of commercial samples of jatobá that should be based on the presence of flavonoids derivatives and low level of procyanidins.

The counterfeiting of the jatobá sap found in this study is possibly related to the difficulty in obtaining this vegetal material, which, besides being produced for only a few months of the year by the plant, as reported by local merchants, is difficult to obtain and can be acquired only when the trunk tree exudes the sap. The simplicity of its production through the decoction of the bark and its reddish color similar to the sap favors the counterfeiting of these samples for the market. This study demonstrates that these two products have a different composition of secondary metabolites, which may result in the absence of the expected effects, yet still bringing side effects to the patient.

Five out of six commercial samples were microbiologically contaminated. Fourteen microorganisms were isolated, eight of which were bacteria (four* Enterobacter* spp., three* Klebsiella* spp., and one* Bacillus s*p.) and six were fungi (four* Candida* spp., one* Rhodotorula* sp., and one* Sporobolomyces salmonicolor*). Four of the five positive cultured saps contained two or more microorganisms within the same sample (Table 3). Microbial contamination was considered high, ranging from 1,700 to 100,000 CFU/mL of the sap.

It has already been established throughout the referential literature that medicinal herbs usually contain bacteria and fungi in their constitution, originating from soil or present in their natural microflora [25]. For the analysis of the CFUs obtained from plant material, the limits set by the Pharmacopeia take into consideration such intrinsic microflora.

According to the World Health Organization [25] plants consumed by the population must be free of* Salmonella sp., Escherichia coli* (<10 CFU), other species of Enterobacteriaceae (<10^3^ CFU), and of yeasts and molds (<10^3^ CFU).

Despite the absence of* Salmonella sp. and Escherichia coli,* the isolation of other enterobacteria such as* Enterobacter* spp. and* Klebsiella* spp. may suggest additional fecal contamination. The lack of hygiene in handling commercial sap may have favored the isolation of these microorganisms. Previous studies have reported that hygiene standards must be established during the cultivation and harvesting of the plant species to avoid cross-contamination [34]. The liquid form of the jatobá sap and its maintenance at room temperature during storage favored the multiplication of microorganisms.

Microbial growth was also observed in Hs-1 (control sample); however, it was considered low in number (of 200 CFU). The bacteria were found to belong to the genus* Bacillus*, which, except* B. anthracis* and* B. cereus* both of which are pathogenic and were not found in this sample, are considered to be environmental contaminants [24].

To summarize, the microbiological contamination found in this study was higher than that permitted by the World Health Organization, making the samples unfit for consumption.

Thus, we issue a warning with regard to the adoption of stricter measures of quality control, from the acquisition of the sap until it is offered for sale. Folk wisdom alone cannot be considered as the single criterion for the consumption of medicinal plants [30].

Another essential factor in the safe use of jatobá sap is the evaluation of toxicity. A plant species exhibiting toxicity can trigger tissue damage to vital organs, causing death in extreme cases due mainly to its low therapeutic index. Thus the toxicity test is an essential step during the analysis of natural products [35].

During the acute toxicity test of the jatobá sap, death was not observed in any of the animals. As for systemic behavioral observations relating to toxicity, except for urinary output, the parameters were considered to be normal and remained unchanged throughout the experiment. No impact on the consciousness of animals or change in general activity or motor coordination, reflexes, or any activity related to the central or autonomous nervous system, indicative of toxicity, was observed. The consumption of food and water was monitored over 14 days and remained without significant change.

The weight of the animals did not change significantly throughout the test. Its observation also can be applied to the relative weights of the kidney and liver after euthanasia when compared to the control group. It is common to find weight change as being a manifestation of toxicity [36].

The hematological and biochemical rates in the treated rats are significantly affected when exposed to potentially toxic compounds [37], and the results obtained, except glucose levels, showed no significant difference (Table 4).

For the acute and subacute toxicity evaluations performed on the leaf extract of* Herniaria glabra*, researchers found a significant decrease in glucose levels of 30% in normoglycemic rats and reported that new studies are being developed in order to determine the hypoglycemic activity of extracts of this plant in a standard test performed on diabetic rats [38]. In this work, as shown in Table 4, when compared to the control group, the groups treated with Hs-1 significantly decrease the rate of glucose in 43% when compared to the normoglycemic rats. This change may indicate a hypoglycemic potential of the plant sample.

It was also observed that the treated rats decreased the frequency of urination throughout the experiment after the administration of the sap by gavage. The control group showed an average of 1.760 ± 1.46 wells during the 14 days of the experiment; the group treated with a 1000 mg/kg dose showed an average frequency of 0.489 wells ± 0.32 (*p* < 0.001) and more concentrated doses, 0.323 ± 0.24 and 0.214 ± 0.44 for 2500 mg/kg and 5000 mg/kg, respectively (*p* < 0.0001), compared with the control group.

The decrease in diuresis may be associated with the decreased of glucose levels, taking into account the fact that hyperglycemia causes a phenomenon known as polyuria (increased urination) and is accentuated due to osmotic diuresis caused by high concentration of glucose in plasma [39]. As the jatobá sap decreased the rate of glucose in normoglycemic rats, the opposite effect could have been accentuated, had the plasma glucose concentration rates been lower.

**Figure 5:**
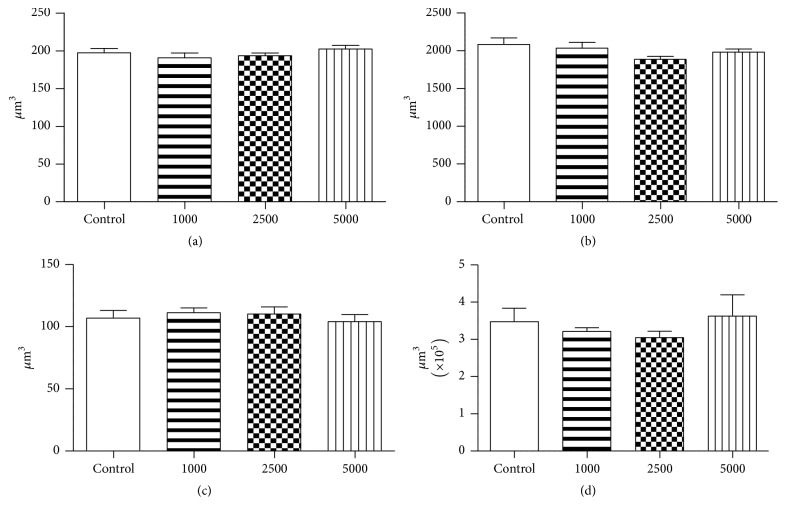
Results expressed by ANOVA followed by the Tukey test. (a) Nuclear volume of hepatocytes. (b) Cell volume of hepatocytes. (c) Cell volume of the proximal convoluted tubule. (d) Volume of the renal glomerulus. Control: rats injected with water; 1000: dose of 1000 mg/kg; 2500: dose of 2500 mg/kg; and 5000: dose of 5000 mg/kg of* H. stigonocarpa* (Hs-1). Values expressed as mean of volume ± error according to average standards.

Regarding the histopathological analysis, no changes in cell structure or any leukocyte infiltration, sinusoidal congestion, or small fat droplets were observed. Quantitatively there were no morphological changes (Figure 5), and the rates of ALT and AST remained unchanged (Table 4).

The kidney, another target for numerous toxic substances, which exhibits a high rate of perfusion and ability to focus on several compounds in the tubular lumen [36], macroscopically showed no renal disorder in this study. There was no difference in the relative weight of the organ, and the morphometric analysis showed no change in nuclear volume of the proximal convoluted tubule and the diameter of the glomerulus (Figure 5), suggesting that tissue integrity of the kidney was preserved and creatinine and urea rates confirmed this observation (Table 4).

The present study demonstrated that 83.3% of samples were considered unsuitable for consumption. The methods used to analyze the authenticity were effective, and the results revealed that none of the commercial samples were obtained from authentic jatobá sap, probably achieved by a decoction of the stem bark or other sources. The commercial samples showed a high microbial contamination and acute toxicity analysis of an authentic sample showed no signs of toxicity and no histological changes in the liver and kidney tissue.

Finally, the evaluated commercial samples do not meet the minimum quality criteria for consumption and may present a danger to people. Therefore, this is an alert to the supervising authorities, to search for new methodologies and ways of implementing more efficient measures to ensure the safety of the population.

## Figures and Tables

**Table 1:** Identification and localization of the studied material.

Code	Material (vegetal part)	Localization (place, state, country)
Js-1	Jatobá commercial (sap)	Campo Grande, MS, Brazil
Js-2	Jatobá commercial (sap)	Campo Grande, MS, Brazil
Js-3	Jatobá commercial (sap)	Campo Grande, MS, Brazil
Js-4	Jatobá commercial (sap)	Campo Grande, MS, Brazil
Js-5	Jatobá commercial (sap)	Campo Grande, MS, Brazil
Js-6	Jatobá commercial (sap)	Campo Grande, MS, Brazil
Hm-1	*Hymenaea martiana *(sap)	Serra do Amolar, MS, Brazil
Hs-1	*Hymenaea stigonocarpa *(sap)	Serra do Amolar, MS, Brazil
Hs-2	*Hymenaea stigonocarpa *(sap)	Serra do Amolar, MS, Brazil
Hm-sb1	*Hymenaea martiana *(stem bark)	Serra do Amolar, MS, Brazil
Hs-sb1	*Hymenaea stigonocarpa *(stem bark)	Serra do Amolar, MS, Brazil
Hs-sb2	*Hymenaea stigonocarpa *(stem bark)	Serra do Amolar, MS, Brazil

**Table 2:** The major compounds present in the sap of *Hymenaea stigonocarpa *(Hs-1) and stem bark (Hs-sb1).

Peak	Time (min)	Compound name	M/S (*m/z*)	Molecular formula	Error (ppm)	Sap/stem bark
1	3.1	Unknown	297.0611	C_13_H_12_O_8_	1	Sap
2	5.2	Unknown	269.0021	—		Stem bark
3	11.0	Unknown	329.0872	C_14_H_16_O_9_	1	Sap
4	19.4	Procyanidin II Derivative	579.1475 579.1491	C_30_H_26_O_12_	5 2	Sap Stem bark
5	21.5	Procyanidin II Derivative glycosyl	725.2069	C_36_H_36_O_16_	3	Stem bark
6	21.1	Flavan-3-ol derivative	275.0910	C_15_H_14_O_5_	4	Sap
7	22.5	Catechin	291.0863 291.0864	C_15_H_14_O_6_	2 2	Sap Stem bark
8	22.9	Procyanidin III Derivative	867.2103	C_45_H_38_O_18_	4	Stem bark
9	24.9	Procyanidin II Derivative	725.2074	C_36_H_36_O_16_	2	Stem bark
10	26.2	Unknown	453.2501	C_24_H_36_O_8_	3	Sap
11	26.2	Unknown	209.1152	—		Sap
12	27.1	Flavonoid Derivative	285.0749	C_16_H_12_O_5_	5	Sap
13	27.2	Unknown	453.2487	C_24_H_36_O_8_	1	Sap
14	27.2	Unknown	209.1152	—		Sap
15	28.5	Unknown	209.0878	—		Sap
16	28.5	Procyanidin II Derivative	563.1534	C_30_H_26_O_11_	2	Sap
17	28.8	Procyanidin II Derivative	579.1496	C_30_H_26_O_12_	2	Stem bark
18	30.0	Flavonol Glycosyl	479.1188	C_22_H_22_O_12_	1	Sap
19	30.4	Unknown	739.1850	C_43_H_30_O_12_	5	Stem bark
20	30.9	Procyanidin II Derivative	563.1553	C_30_H_26_O_11_	1	Sap
21	31.1	Epicatechin	291.0858 291.0857	C_15_H_14_O_6_	4 4	Sap Stem bark
22	32.1	Flavonoid Derivative	289.0698	C_15_H_12_O_6_	5	Sap
23	32.1	Unknown	271.0599	C_15_H_10_O_5_	3	Sap
24	32.3	Procyanidin II Derivative	723.1900	C_36_H_34_O_16_	4	Stem bark
25	32.9	Unknown	739.1832	C_43_H_30_O_12_	3	Stem bark
26	32.9	Flavanonol Derivative	319.0801	C_16_H_14_O_7_	5	Sap
27	35.3	Procyanidin II Derivative	563.1541	C_30_H_26_O_11_	2	Sap
28	35.5	Procyanidin II Derivative	723.1904	C_36_H_34_O_16_	3	Stem bark
29	36.4	Unknown	739.1847	C_43_H_30_O_12_	4	Stem bark
30	36.7	Procyanidin III Derivative	867.2092	C_45_H_38_O_18_	5	Stem bark
31	37.9	Procyanidin II Derivative	563.1541	C_30_H_26_O_11_	3	Sap
32	38.9	Procyanidin II Derivative	723.1901	C_36_H_34_O_16_	4	Stem bark
33	40.2	Flavanonol Derivative	319.0802	C_16_H_14_O_7_	5	Sap
34	40.5	Unknown	277.1060	C_15_H_16_O_5_	6	Sap
35	40.9	Flavone Derivative	285.0753	C_16_H_12_O_5_	5	Sap
36	41.2	Flavan-3-ol Derivative	305.1019	C_16_H_16_O_6_	2	Sap
37	41.2	Unknown	153.0534	—		Sap
38	41.7	Procyanidin II Derivative	563.1542	C_30_H_26_O_11_	3	Sap
39	41.9	Procyanidin II Derivative	723.1911	C_36_H_34_O_16_	2	Stem bark
40	42.4	Procyanidin II Derivative	563.1553	C_30_H_26_O_11_	1	Sap
41	42.9	Unknown	739.1844	C_43_H_30_O_12_	4	Stem bark
42	43.6	Unknown	739.1851	C_43_H_30_O_12_	5	Stem bark
43	44.8	Procyanidin II Derivative	563.1533	C_30_H_26_O_11_	4	Sap
44	47.3	Unknown	249.1167 249.1149	—	—	Sap Stem bark
45	47.4	Procyanidin II Derivative	563.1545	C_30_H_26_O_11_	2	Sap
46	47.7	Flavanonol Derivative	305.0647	C_15_H_12_O_7_	5	Sap
47	48.3	Procyanidin II Derivative	563.1567	C_30_H_26_O_11_	3	Sap
48	50.0	Procyanidin II Derivative	563.1561	C_30_H_26_O_11_	2	Sap
49	50.6	Unknown	153.0538	—		Sap
50	51.1	Flavanonol Derivative	333.0974	C_17_H_16_O_7_	1	Sap
51	51.3	Flavanonol Derivative	305.0666 305.0667	C_15_H_12_O_7_	2 2	Sap Stem bark
52	51.6	Flavonoid Derivative	287.0551 287.0550	C_15_H_10_O_6_	2 2	Sap Stem bark
53	51.7	Procyanidin II Derivative	563.1555	C_30_H_26_O_11_	1	Sap
54	51.7	Flavonoid Derivative	331.0823	C_17_H_14_O_7_	2	Sap
55	51.8	Flavan-3-ol Derivative	305.1011	C_16_H_16_O_6_	5	Sap
56	52.0	Flavonoid Derivative	301.0709	C_16_H_12_O_6_	2	Sap
57	52.1	Flavanonol Derivative	319.0818	C_16_H_14_O_7_	1	Sap
58	52.2	Flavone Derivative	345.0979	C_18_H_16_O_7_	2	Sap
59	52.4	Quercetin	303.0513	C_15_H_10_O_7_	3	Sap
60	52.4	Flavone Derivative	373.1287 373.1289	C_20_H_20_O_7_	1 1	Sap Stem bark
61	52.8	Unknown	305.2480	C_20_H_32_O_2_	1	Sap
63	53.8	Unknown	192.1406	—		Stem bark

**Table 3:** Identification of bacteria and fungi present in the commercial samples (Js-1 to Js-6) and control sap (Hs-1).

Sample	Growth	Morphology	Identification
Js-1	2,550 CFU ± 220	GPB	*Bacillus *sp.
Js-1	2,200 CFU ± 500	Yeast	*Candida lusitaniae*
Js-3	≥100,000 CFU	GNB	*Enterobacter gergoviae*
Js-3	≥100,000 CFU	GNB	*Klebsiella oxytoca*
Js-3	1,700 CFU ± 60	Yeast	*Candida albicans*
Js-3	34,000 CFU ± 300	Yeast	*Rhodotorula *sp.
Js-4	≥100,000 CFU	GNB	*Enterobacter gergoviae*
Js-4	≥100,000 CFU	GNB	*Klebsiella oxytoca*
Js-4	55,000 CFU ± 500	Yeast	*Sporobolomyces salmonicolor*
Js-4	≥100,000 CFU	Yeast	*Candida tropicalis*
Js-5	61,100 CFU ± 1100	GNB	*Enterobacter cloacae*
Js-5	11,900 CFU ± 570	GNB	*Klebsiella oxytoca*
Js-5	≥100,000 CFU	Yeast	*Candida tropicalis*
Js-6	61,000 UFC ± 800	GNB	*Enterobacter agglomerans*
Hs-1	200 UFC ± 40	GPB	*Bacillus *sp.

GPB: Gram positive bacilli; GNB: Gram negative bacilli; CFU: colony forming unit.

**(a):** 

*Haematological parameters*	*Control *(n = 5)	*T1 *(n = 9)	*T2 *(n = 9)	*T3 *(n = 12)
RBC (10^6^/Mm^3^)	7.858 ± 0.28	8.45 ± 0.72	8.19 ± 0.51	8.47 ± 0.54
Hemoglobin (g/dL)	14.54 ± 0.38	15.44 ± 1.24	15.07 ± 0.77	15.39 ± 0.91
Hematocrit (%)	45.04 ± 1.48	49.81 ± 5.35	47.58 ± 2.69	49.75 ± 3.05
MCV (fL)	57.36 ± 1.86	58.98 ± 3.99	58.13 ± 1.54	58.77 ± 2.27
MCH (pg)	18.52 ± 0.31	18.31 ± 0.64	18.41 ± 0.69	18.17 ± 0.44
MCHC (g/dL)	32.3 ± 0.67	31.09 ± 1.51	31.70 ± 1.07	30.96 ± 0.89
Platelets (10^3^/Mm^3^)	489.8 ± 102.83	603.56 ± 151.58	557.33 ± 71.77	593.92 ± 173.49
Leukocytes (10^3^/Mm^3^)	7.68 ± 2	10.06 ± 4.44	9.45 ± 1.75	9.56 ± 4.40
Neutrophils (%)	22.4 ± 5.8	16.78 ± 4.61	14.70 ± 3.47	14.08 ± 3.83
Lymphocytes (%)	72.45 ± 9.21	76.98 ± 5.22	79.29 ± 5.59	80.63 ± 6
Monocytes (%)	3.02 ± 1.52	4.02 ± 2.02	4.35 ± 2.47	3.63 ± 2.81
Eosinophils (%)	2 ± 3.60	1.67 ± 2.02	1.03 ± 0.57	1.02 ± 0.43
Basophils (%)	0.12 ± 0.09	0.56 ± 0.64	0.61 ± 0.34	0.65 ± 0.39

**(b):** 

*Biochemical parameters*	*Control *(n = 8)	*T1 *(n = 12)	*T2 *(n = 12)	*T3 *(n = 10)
Urea (mg/dL)	47 ± 6.48	49.33 ± 5.82	46.25 ± 4.67	51 ± 6.80
Creatinine (mg/dL)	0.4 ± 0.06	0.47 ± 0.12	0.45 ± 0.05	0.42 ± 0.04
Amylase (U/L)	2360 ± 375.14	342.67 ± 305.27	2376.92 ± 235	2374.80 ± 451.59
Lipase (U/L)	5.75 ± 0.31	6.53 ± 0.69	6.46 ± 0.68	6.28 ± 0.37
AST (U/L)	135.5 ± 90.01	185.42 ± 107.29	159.17 ± 79.26	115 ± 38.11
ALT (U/L)	40.5 ± 15.92	63.92 ± 13.64	63.58 ± 23.04	47.60 ± 10.90
Glucose (mg/dL)	269 ± 57.28	157 ± 34.03^*∗∗∗*^	162.75 ± 62.17^*∗∗∗*^	139 ± 36.45^*∗∗∗*^

Data expressed as mean average ± standard deviation. Control: rats treated with water T1: dose of 1000 mg/kg, T2: dose of 2500 mg/kg, and T3: dose of 5000 mg/kg of the sap of the *Hymenaea stigonocarpa *(Hs-1). MCV: mean corpuscular volume, MCH: mean corpuscular haemoglobin, MCHC: mean corpuscular haemoglobin concentration, ALT: alanine aminotransferase, and AST: aspartate aminotransferase. ^*∗∗∗*^*p* ≤ 0.001 (compared with the control group).
